# Multiple gene-deletion vaccinia virus Tiantan strain against mpox

**DOI:** 10.1186/s12985-025-02629-6

**Published:** 2025-01-25

**Authors:** Yilong Zhu, Dapeng Li, Renshuang Zhao, Minghua Chen, Yue Li, Xia Yang, Hongyue Mao, Xiao Li, Yiquan Li, Chao Shang, Xianzhu Xia

**Affiliations:** 1 Changchun Veterinary Research Institute, Chinese Academy of Agricultural Sciences, Changchun, 130122 People’s Republic of China; 2 Key Laboratory of Jilin Province for Traditional Chinese Medicine Prevention and Treatment of Infectious Diseases, College of Integrative Medicine, Changchun University of Chinese Medicine, Changchun, 130117 People’s Republic of China

**Keywords:** Attenuated vaccinia virus, Monkeypox virus, Mouse and rabbit models, Immunogenicity and protective efficacy

## Abstract

Monkeypox virus (MPXV) is an important zoonotic pathogenic virus, which poses serious threats to public health. MPXV infection can be prevented by immunization against the variola virus. Because of the safety risks and side effects of vaccination with live vaccinia virus (VACV) strain Tian Tan (VTT), we constructed two gene-deleted VTT recombinants (TTVAC7 and TTVC5). The immunogenicity and protective effects of the gene-deleted VTT vaccine were assessed using BALB/C mice challenged with VTT and New Zealand rabbits challenged with MPXV. The results demonstrated strong humoral and cellular immune responses. The VTT-specific and neutralizing antibody titers, specific T cell levels, and degree of dendritic cell maturation of the mice, in addition to the MPXV neutralizing antibody titers and IFN-γ, IL-6, and TNF-α levels of the rabbits were markedly higher in the groups immunized with TTVAC7 and TTVC5 than the control groups (*p* < 0.05). Moreover, immunization with TTVAC7 and TTVC5 reduced morbidities caused by VACV and MPXV infection. The weight change, lung histological score, and residual virus of the mouse model (*p* < 0.05). Similarly, the temperature change, pock number, lung histological score, and residual virus of the rabbit model were significantly reduced in the groups immunized with TTVAC7 and TTVC5 (*p* < 0.05). Collectively, these results demonstrate that TTVAC7 and TTVC5 may be used as potential live attenuated vaccines against MPXV infection.

## Introduction

Monkeypox virus (MPXV), the causative agent of mpox infection, can cause smallpox-like clinical symptoms, including skin rash, fever, and headache [1], and currently presents a global health emergency of international concern [2, 3]. Although there is no specific vaccine against mpox, the smallpox vaccine can provide cross-protection [4–6] and is reportedly 85% effective against MPXV [7]. In China, the vaccine strain VTT was isolated from the smallpox scabs of a soldier in February 1926 [8, 9]. To test the infectivity and potential as a vaccine, a monkey was cutaneously inoculated with a slurry of the blisters of the soldier, which resulted in an active infection. Then, another monkey was infected and the blister plasma obtained from the monkey was inoculated on the skin and testicles of rabbits for five consecutive generations, and then transferred to the skin of calves for three consecutive generations.

The vaccine strain VTT not only prevented infection of the variola virus but also caused five human infections, which included one case of severe pneumonia and four cases of skin rashes in Wuxi (Jiangsu, China) in March 2017 [10]. Moreover, the vaccinia virus-related smallpox vaccine was shown to induce severe adverse reactions in immunocompromised individuals, such as encephalitis and myocarditis [11–13].

Our group previously, reported the development of two gene-deleted VTT vaccines using homologous recombination and the Cre/Loxp system, including TTVAC7 (deleted genes: TC7L-TK2L, TE, TI4L, TJ2R, TA35R, TA66R, and TB13R) and TTVC5 (deleted genes: TC7L-TK2L, TE, TA35R, TA66R, and TB13R), which exhibited attenuated virulence as compared to VTT [14, 15]. The deleted fragments were related to virulence (TC7L-TK2L, TE, TA35R, TA66R, TB13R, and TI4L), the host (TC7L-TK2L, and TE), and immunomodulation (TC7L-TK2L, TE, TB13R, and TA35R).

Although TTVAC7 and TTVC5 have shown superior safety as compared to VTT [14, 15], the ability of TTVAC7 and TTVC5 to prevent MPXV infection remains unclear. Therefore, BALB/C mice challenged with VTT and New Zealand rabbits challenged with MPXV were used to evaluate the protective effects of TTVAC7 and TTVC5 against MPXV infection.

## Materials and methods

### Reagents, viruses, and animals

A mouse spleen lymphocyte isolation kit was obtained from Tianjin Haoyang Biological Manufacture Co., Ltd. (Tianjin, China). Cell staining buffer, phycoerythrin (PE)-conjugated anti-mouse IL-4 and IFN-γ antibodies, allophycocyanin (APC)-conjugated anti-mouse CD3 antibody, fluorescein isothiocyanate (FITC)-conjugated anti-mouse CD4 antibody, peridinin chlorophyll protein complex (PerCP)-conjugated anti-mouse CD8 antibody, FITC-conjugated anti-mouse CD11c antibody, and PE-conjugated anti-mouse CD80/CD86/CD40/MHC II were obtained from BioLegend (San Diego, CA, USA). A fixation/permeabilization kit was obtained from BD Biosciences (Franklin Lakes, NJ, USA). Rabbit IFN-γ, IL-6, and TNF-α enzyme-linked immunosorbent assay (ELISA) kits were obtained from Jiangsu Meibiao Biotechnology Co., Ltd. (Jiangsu, China).

VTT (GenBank: AF095689.1), TTVAC7, TTVC5, and MPXV (West African strain, GenBank: PP778666.1) were stored at Changchun Veterinary Research Institute, Chinese Academy of Agricultural Sciences (Changchun, China).

Female BALB/C mice (age, 6 weeks) and male New Zealand rabbits (age, 2 months) were supplied by SiPeiFu Biotechnology Co., Ltd. (Beijing, China). All surgeries were performed under sodium pentobarbital anesthesia and all efforts were made to minimize suffering.

### Mice and rabbit vaccination

Female BALB/C mice were divided into five groups (*n* = 10) and intramuscularly immunized with VTT, TTVAC7, or TTVC5 at 1 × 10^6^ plaque-forming units (PFU) in 200 µL of normal saline. The control and model groups were injected with 200 µL of normal saline. Serum samples were collected on day post-immunization (dpi) 28 for detection of neutralizing antibody titers of VTT. The control group did not receive immunized and challenged, while the model group was unimmunized but received challenged.

Male New Zealand rabbits were divided into five groups (*n* = 5) and subcutaneously immunized with VTT, TTVAC7, or TTVC5 at 10^6^ PFU in 200 µL of normal saline. The control and model groups were injected with 200 µL of normal saline. Serum samples were collected on dpi 7, 14, 21, and 28 for detection of neutralizing antibody titers of MPXV. The control group did not receive immunized and challenged, while the model group was unimmunized but received challenged.

### Flow cytometry analysis

Briefly, mouse splenocytes (3 × 10^6^) were collected on dpi 28 using a mouse spleen lymphocyte isolation kit and stimulated with inactivated VTT at 1.5 × 10^4^ PFU in 15 µL of normal saline for 7 days at 37 °C under an atmosphere of 5% CO_2_. Afterward, the cells were collected, washed once with cell staining buffer, and stained with fluorescent antibodies (CD3/APC, CD4/FITC, and CD8/PerCP) for 30 min at 4°C in the dark. After fixation and permeabilization, the splenocytes were washed once and divided into two groups, which were stained with antibodies against IFN-γ/PE and IL-4/PE, respectively, for 30 min at 4°C in the dark. The expression levels of CD3^+^CD4^+^/IFN-γ/IL-4 and CD3^+^CD8^+^/IFN-γ/IL-4 were determined by flow cytometry.

Meanwhile, mouse dendritic cells (5 × 10^6^) were isolated from the femur and tibia of mice on dpi 28 and stimulated with inactivated VTT at 1.5 × 10^4^ PFU in 15 µL of normal saline for 7 days at 37 °C under an atmosphere of 5% CO_2_. Afterward, the cells were collected, washed once with cell staining buffer, and incubated with fluorescent antibodies (CD11c/FITC) for 30 min at 4°C in the dark. Then, the dendritic cells were washed once and divided into four groups, which were stained with antibodies against CD80/PE, CD86/PE, CD40/PE, and MHC II/PE, respectively, for 30 min at 4°C in the dark. The proportions of CD11c^+^, CD80^+^, CD86^+^, CD40^+^, and MHC II^+^ cells were determined by flow cytometry (Accuri™ C6 Plus; BD Biosciences).

### ELISA analysis

The titers of VTT-specific immunoglobulin (Ig) G were detected with an ELISA of 2 × 10^5^ PFU/100 µL of VTT-coated 96-well plates. The optical density was measured at 450 nm. The final titers were determined as an absorbance of the maximum serum dilution that was two-fold higher than the background values.

Rabbit serum levels of IFN-γ, IL-6, and TNF-α on dpi 28 were analyzed using ELISA kits in accordance with the manufacturer’s instructions.

### Neutralizing antibody detection

Two-fold dilutions of mouse and rabbit serum were treated with VTT or MPXV at 100 PFU for 1 h at 37 °C. Vero E6 cells were incubated with the VTT or MPXV solution in the wells of 96-well plates for 2 h at 37 °C under an atmosphere of 5% CO_2_. Afterward, the solution was discarded and replaced with 2% Dulbecco’s modified Eagle’s medium and incubation was continued for 72 h at 37 °C under an atmosphere of 5% CO_2_. Neutralizing antibody titers were determined as the reciprocal of the serum dilution that reduced the amount of viral plaques by 50%. The results were calculated using the Reed Muench method [16].

### Virus challenge

On dpi 28, mice were challenged intranasally with VTT at 10^6^ PFU and rabbits were challenged intravenously with MPXV at 10^6.5^ PFU. Weight loss of the infected mice was monitored for 14 days. 3 mice in each group were euthanized on dpi 7 and the partial lung, nose, and trachea were harvested for detection of residual virus. The other lung tissues were stained with hematoxylin and eosin (H&E). The body temperature of the rabbits was measured on dpi 5. The infected rabbits were euthanized and the number of pocks was measured on dpi 7. The lungs and testes were harvested and portioned for detection of residual virus and staining with H&E.

### H&E staining

The lungs of mice and lungs and testes of rabbits were fixed in 4% paraformaldehyde solution and stained with H&E as described previously [17]. Lung histological score were calculated according to the number of neutrophils in the alveolar space (0–2 points) or the lung interstitial space (0–2 points), degree of alveolar epithelial injury (0–2 points), alveolar interstitial thickness (0–2 points), and level of alveolar hemorrhage or erythrocyte extravasation (0–2 points) [18, 19].

### Detection of residual virus

The residual virus of VTT of homogenates of the mouse lung, nose, and trachea were determined using a plaque assay with Vero E6 cells.

MPXV residues in homogenates of rabbit lungs and testes were detected using a plaque assay with Vero E6 cells.

### Statistical analysis

Statistical analyses were performed using Prism 8.0 software (GraphPad Software, LLC, San Diego, CA, USA). The one-way analysis of variance (ANOVA) was used to compare multi-group variables and the data between the two groups were analyzed by Student’s T Test. All the data are presented as the mean ± standard deviation (SD).

## Results

### TTVAC7 and TTVC5 induced strong humoral and cellular immune responses in mice

**Fig. 1:**
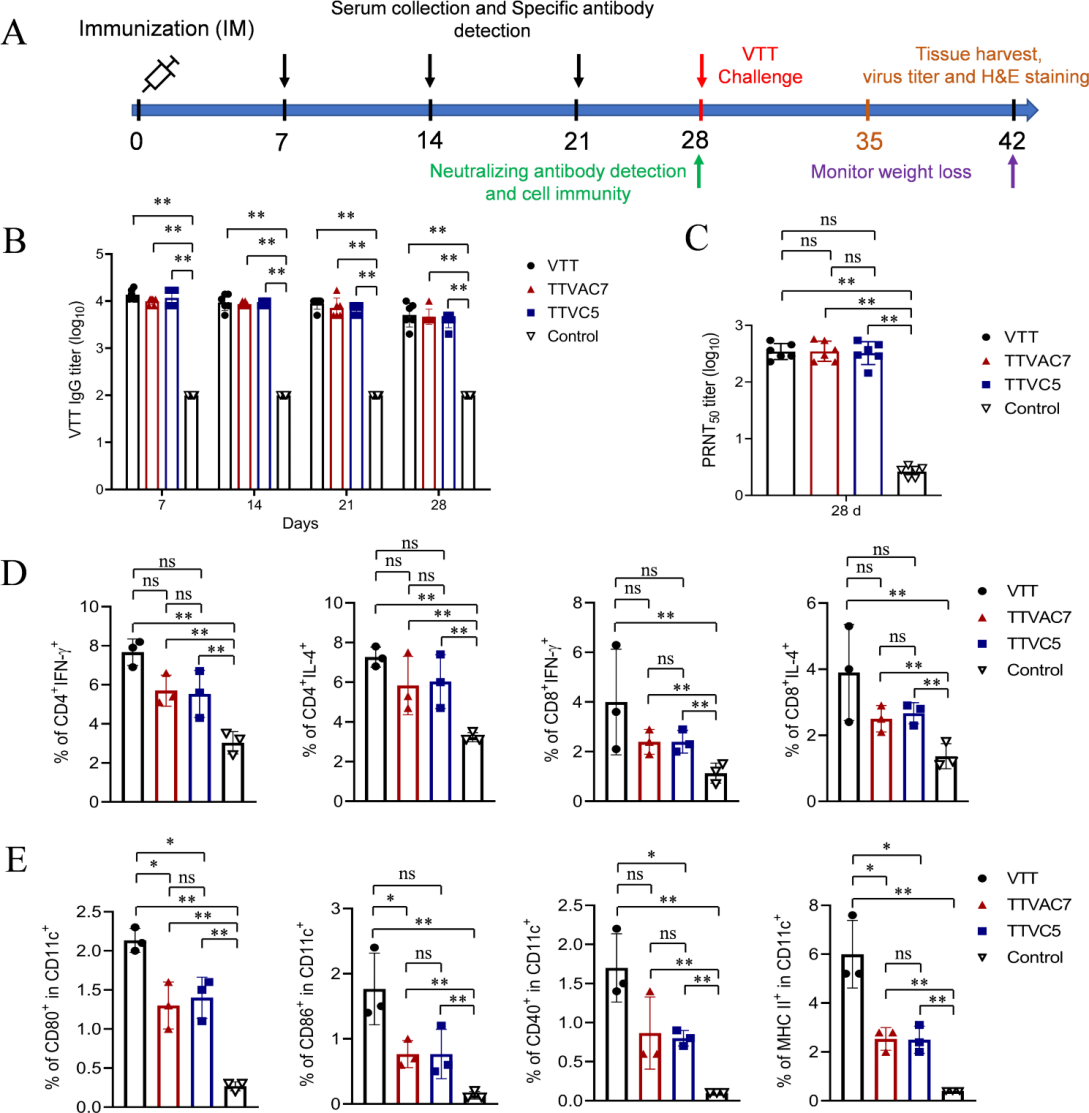
Immune response to TTVAC7 and TTVC5 in immunized mice. BALB/C mice were vaccinated with TTVAC7, TTVC5, and VTT at 10^6^ PFU by intramuscular injection. Serum samples were collected every 7 days for 28 days. Mouse spleen lymphocytes and dendritic cells were separated on dpi 28 for the detection of VTT-specific cellular immune responses. (**A**) The experimental procedure. VTT-specific antibody levels (**B**) and neutralization antibody titers (**C**) were measured with an ELISA and a viral plaque formation assay. The levels of VTT-specific CD3^+^CD4^+^/CD8^+^ cells expressing IFN-γ/IL-4 (**D**) and CD11c^+^/CD80^+^/CD86^+^/CD40^+^/MHC II^+^ (**E**) were detected by flow cytometry (ns, not significant, *p < 0.05, **p < 0.01)

The immunogenicity of TTVAC7 and TTVC5 in mice was evaluated by detection of VTT-specific IgG and neutralizing antibody titers, specific T cell levels, and the degree of dendritic cell maturation. The experimental procedure is shown in Fig. 1A. Serum titers of VTT-specific IgG were remarkably higher in the TTVAC7 and TTVC5 groups than the control groups on dpi 7, 14, 21, and 28 (*p* < 0.01; Fig. 1B). Meanwhile, the titers of VTT neutralizing antibody were markedly higher in the TTVAC7 and TTVC5 immunized groups than the control group on dpi 28 (*p* < 0.01; Fig. 1C).

The levels of CD3^+^CD4^+^/CD8^+^ cells expressing IFN-γ/IL-4 in addition to CD11c^+^/CD80^+^/CD86^+^/CD40^+^/MHC II^ + ^levels were markedly higher in the TTVAC7 and TTVC5 immunized groups than the control group on dpi 28 (*p* < 0.01; Fig. 1D and E). These results indicate that TTVAC7 and TTVC5 induced strong humoral and cellular immune responses in BALB/C mice.

### TTVAC7 and TTVC5 protected BALB/C mice against VTT challenge

**Fig. 2:**
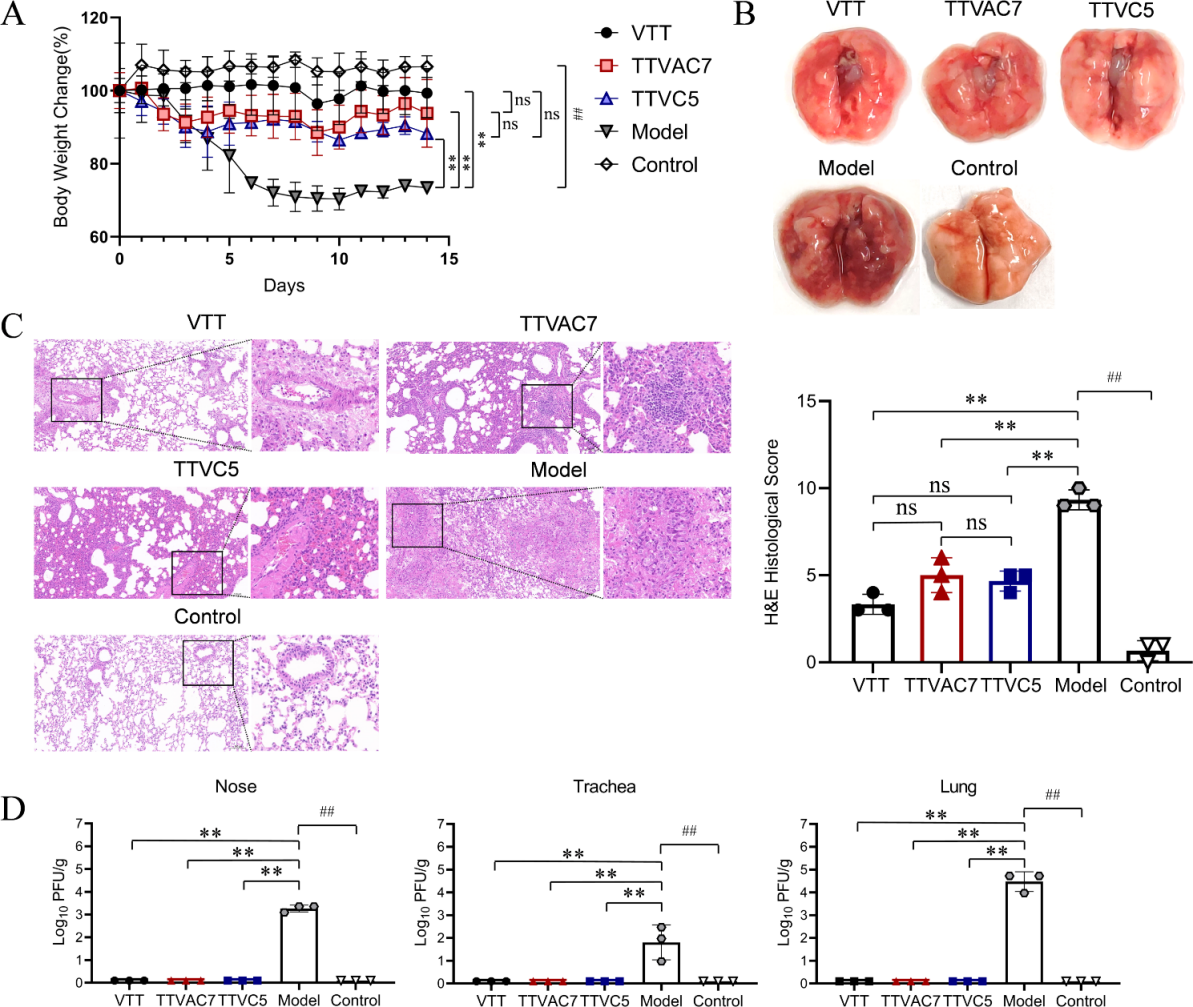
Protective efficiency of TTVAC7 and TTVC5 in mice against VTT challenge. (**A**) Changes to the body weight of mice after VTT challenge. A remarkable weight loss could be observed in the model group. (**B**,** C**) Images of lung tissues, H&E staining, and H&E histological score (magnification of 20× and 63×). The residual virus of mice after VTT challenge were measured with a viral plaque formation assay. (**D**) The VTT titers of nose, trachea, and lung tissues in each group (ns, not significant, **p < 0.01, ^##^*p* < 0.01)

The protective efficacy of TTVAC7 and TTVC5 in BALB/C mice challenged with VTT was estimated by changes to body weight, lung histological score, and residual virus. As shown in Fig. 2A, after VTT challenge, the body weight of mice was remarkably lower in the model group than the TTCAC7 and TTVC5 groups on dpi 6 to 14 (*p* < 0.01).

Moreover, the lung histological score was remarkably lower in the TTVAC7 and TTVC5 groups than the model group on dpi 7 (*p* < 0.01; Fig. 2B, C). H&E staining revealed increased amounts of neutrophils and erythrocytes, damage to the alveolar epithelium, thickening of the alveolar interstitium, and intra-alveolar hemorrhage in the model group.

The results of residual virus are shown in Fig. 2D. VTT titers of the nose, trachea, and lung tissues of mice were remarkably lower in the TTVAC7 and TTVC5 groups than the model group (*p* < 0.01). Notably, infectious virus particles were only detected in the model group on dpi 7. These results show that TVAC7 and TTVC5 protected BALB/C mice against VTT challenge.

### TTVAC7 and TTVC5 induced strong humoral and cellular immune responses in rabbits

**Fig. 3:**
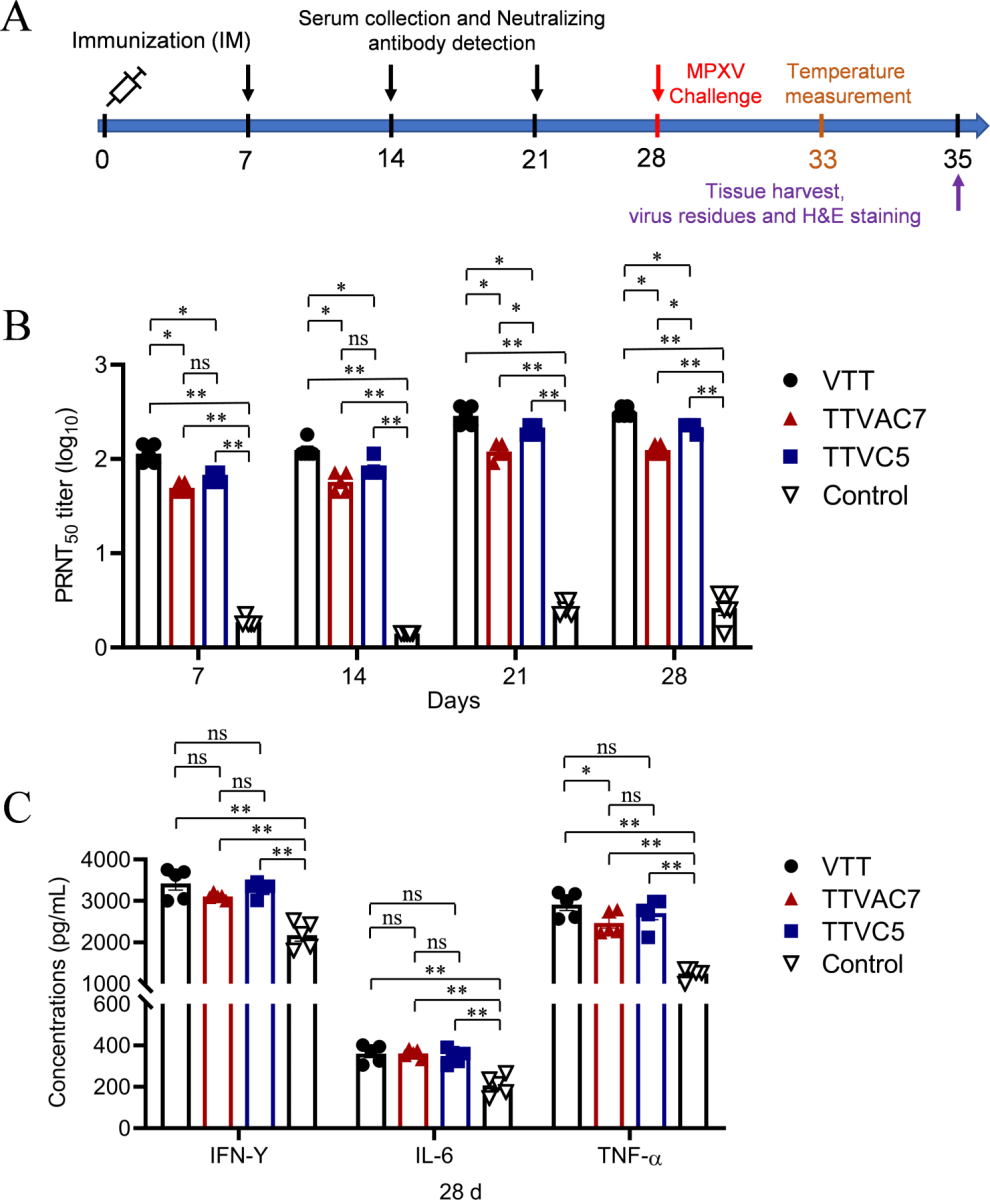
Immune response to TTVAC7 and TTVC5 in immunized rabbits. New Zealand rabbits were vaccinated with TTVAC7, TTVC5, and VTT at 10^6^ PFU by subcutaneous injection. Serum samples were collected every 7 days for 28 days. (**A**) The experimental procedure. The neutralization antibody titers (**B**) and IFN-γ, IL-6, and TNF-α levels (**C**) were measured with a viral plaque formation assay and ELISAs, respectively (ns, not significant, *p < 0.05, **p < 0.01)

The immunogenicity of TTVAC7 and TTVC5 in rabbits was evaluated by detecting MPXV neutralizing antibody titers and IFN-γ, IL-6, and TNF-α levels. The experimental procedure is shown in Fig. 3A. The titers of MPXV neutralizing antibody were remarkably higher in the TTVAC7 and TTVC5 immunized groups than the control group on dpi 7, 14, 21, and 28 (*p* < 0.01; Fig. 3B).

Meanwhile, IFN-γ, IL-6, and TNF-α levels were markedly higher in the TTVAC7 and TTVC5 groups than the control group on dpi 28 (*p* < 0.01; Fig. 3C). These results demonstrate that TTVAC7 and TTVC5 induced strong humoral and cellular immune responses in New Zealand rabbits.

### TTVAC7 and TTVC5 reduced injuries in New Zealand rabbits infected with MPXV

**Fig. 4:**
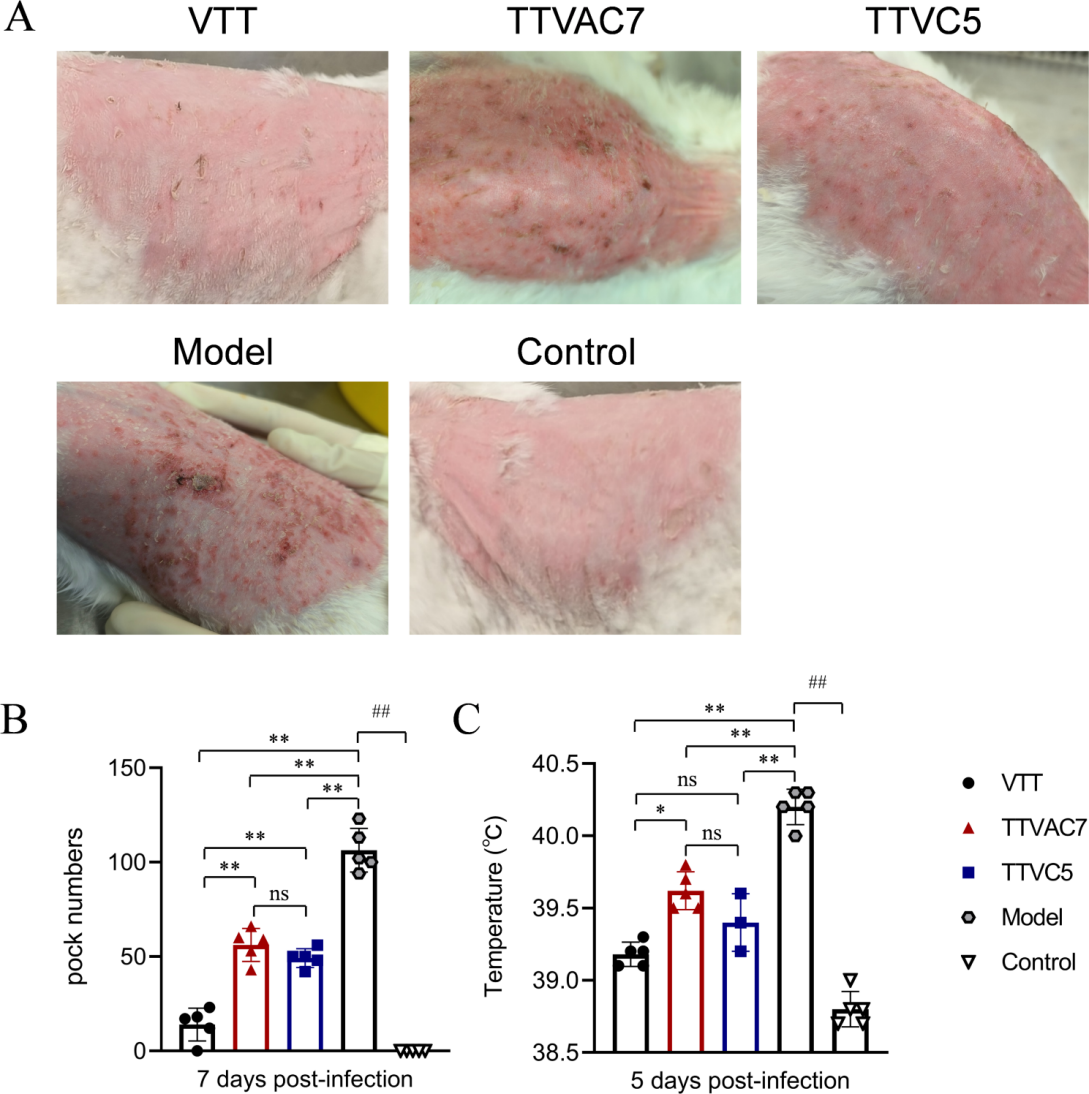
Pock numbers and changes to body temperature. (**A**,** B**) The pock numbers of rabbits were counted on day 7 after challenge with MPXV at 10^6.5^ PFU. The model group had more pocks caused by MPXV. (**C**) Changes to the body temperature of rabbits after MPXV challenge. Low-grade fever was observed in the TTVAC7- and TTVC5-immunized groups, whereas high-grade fever was evident in the model group (ns, not significant, *p < 0.05, **p < 0.01, ^##^*p* < 0.01)

The protective efficacy of TTVAC7 and TTVC5 was evaluated in New Zealand rabbits challenged with MPXV by evaluating the pock numbers, changes to body temperature, histological scores of the lungs and testes, and residual virus. Representative photos of pock lesions of rabbits are shown in Fig. 4A. The number of pocks was greater in the model group than the TTVAC7 and TTVC5 groups on dpi 7 (*p* < 0.05; Fig. 4B).

Body temperature was significantly higher in the model group than the TTVAC7 and TTVC5 groups on dpi 5 (*p* < 0.05) (Fig. 4C).

**Fig. 5:**
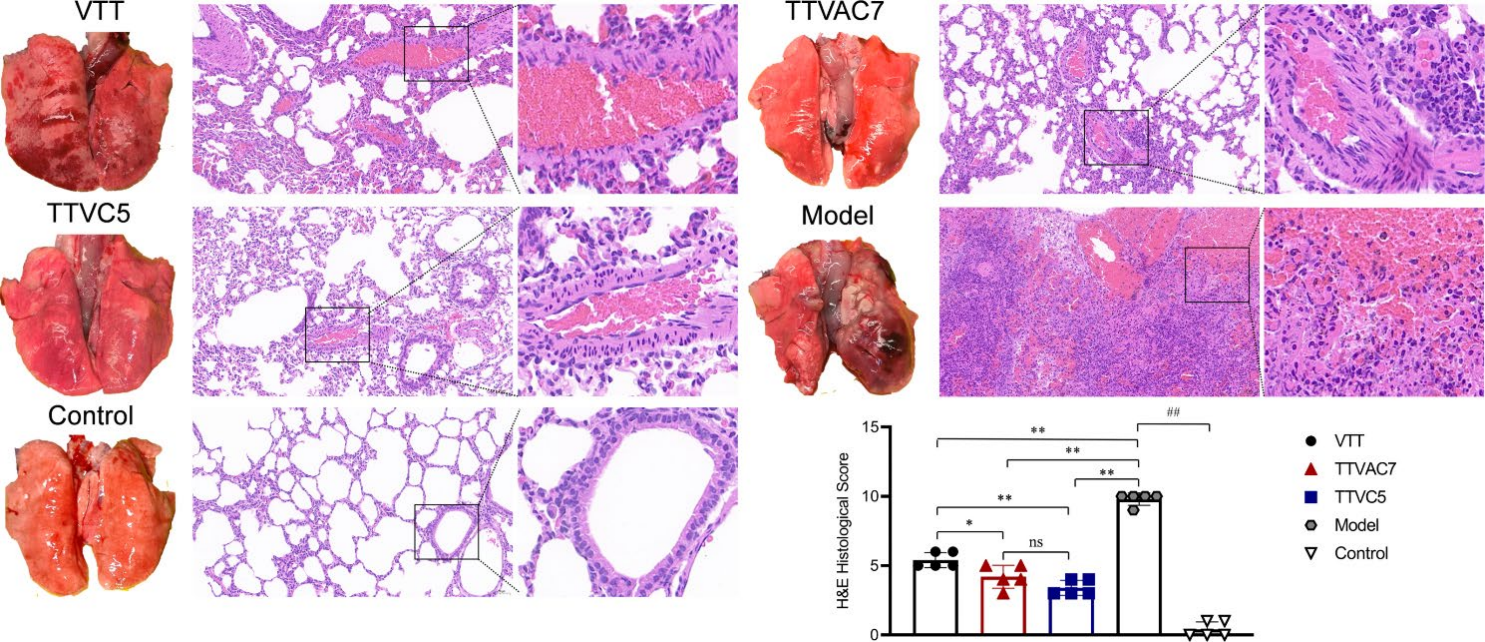
Degree of injury to the lung tissues of rabbits after MPXV challenge. Severe lung injury and histological score were lower in the TTAVC7- and TTAC5-immunized groups than the model group (magnification of 20× and 63×) (ns, not significant, *p < 0.05, **p < 0.01, ^##^*p* < 0.01)

**Fig. 6:**
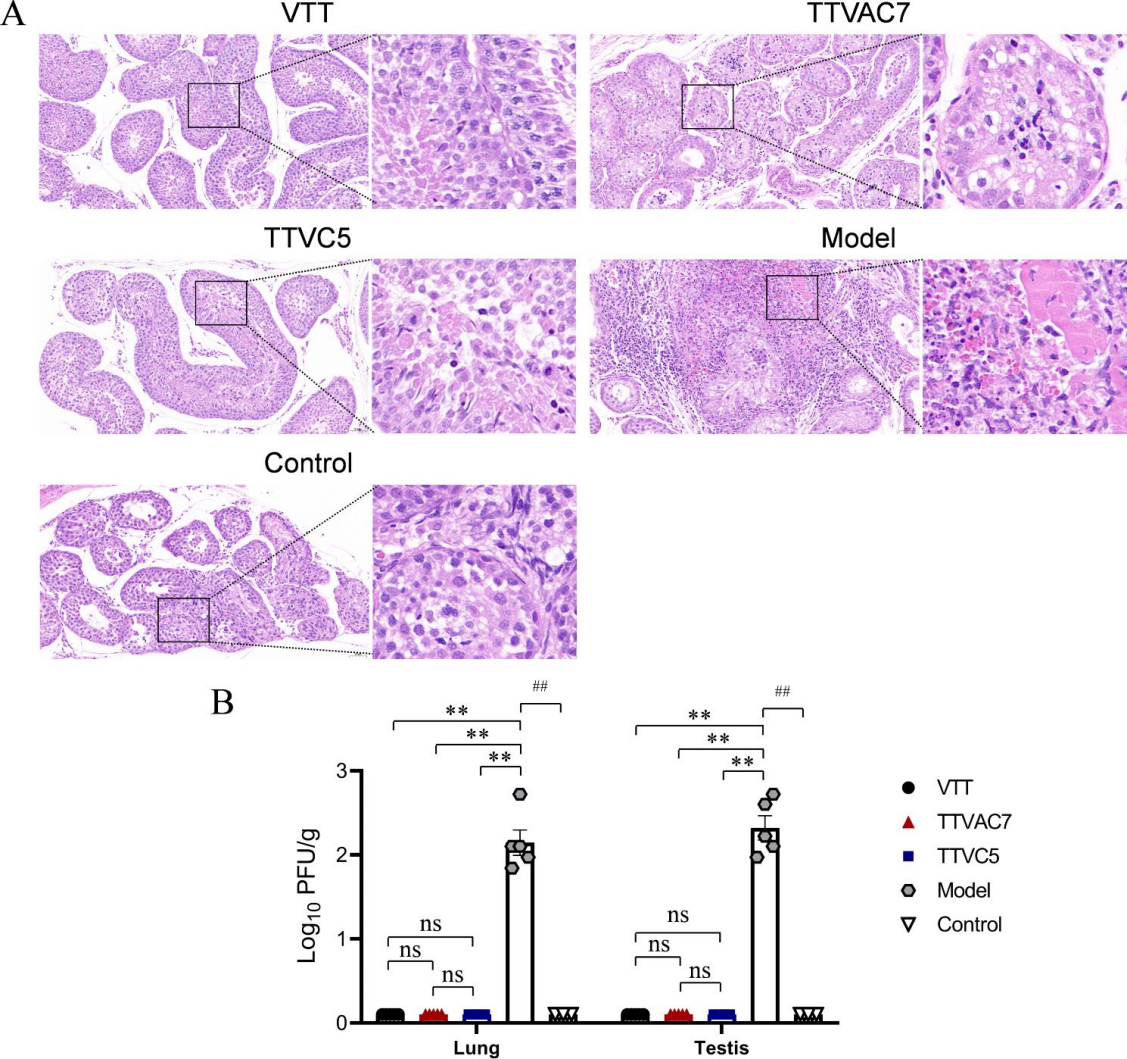
H&E data of testis tissue and viral titers from lung and testis tissues. (**A**) H&E staining of testis tissue on day 7 after MPXV challenge (magnification of 20× and 63×). (**B**) The MPXV titers of lung and testis tissues in rabbits were detected using a viral plaque formation assay. More severe testis injury and higher viral titers were exhibited in the model group than the TTVAC7- and TTVC5-immunized groups (ns, not significant, *p < 0.05, **p < 0.01, ^##^*p* < 0.01)

Moreover, as compared to the TTVAC7 and TTVC5 groups, the lung tissues of the model group had more severe injury, damaged alveolar epithelium, thickened alveolar interstitium, and intra-alveolar hemorrhage as well as greater numbers of neutrophils and erythrocytes, on dpi 7 (Fig. 5). H&E straining illustrated that injury to the testes was more severe in the model group than the TTVAC7 and TTVC5 groups on dpi 7. Extensive necrosis and focal hemorrhage and large numbers of infiltrating inflammatory cells were clearly observed in the model group (Fig. 6A).

The results of residual virus are shown in Fig. 6B. The titers of MPXV in the lungs and testes was remarkably higher in the model group than the TTVAC7 and TTVC5 groups (*p* < 0.01). These results show that TTVAC7 and TTVC5 could reduce injuries of New Zealand rabbits infected with MPXV.

## Discussion

Among double-positive CD3^+^CD4^+^ helper T cells, Th1 cells are differentiated by stimulation with IL-12, IFN-γ, and IL-2, and express the cytokines IFN-γ, TNF-α, and IL-2; stimulate cellular immunity, promote activation of macrophages and monocytes, and regulate T lymphocyte-mediated cytotoxicity and cellular immunity, demonstrating the important roles of Th1 cells in clearing intracellular pathogenic microbes [20, 21]. Among double-positive CD3^+^CD8^+^ cytotoxic T lymphocytes, Tc1 cells are differentiated by stimulation with IL-12 and IL-2, and express the cytokines IFN-γ and TNF-α to kill target cells [22], while Tc2 cells are differentiated by stimulation with IL-4 and express the cytokine IL-4, and activate CD4^+^ T cells, which participate in immune responses [23]. IFN-γ and IL-4 reportedly play important roles in immune reactions against vaccinia virus infection in mice [24].

CD80 and CD86 are costimulatory proteins expressed on the surfaces of antigen-presenting cells (i.e., dendritic cells, B cells, and monocytes) and provide costimulatory signals to T cells by interacting with CD28 and CTLA4 [25, 26]. MHC class II molecules, which are expressed on the surfaces of antigen-presenting cells (i.e., macrophages, dendritic cells, and B cells) provide antigens to helper T cells to initiate the immune response [27, 28]. The CD40 receptor and its ligand CD40L belong to the TNF/TNFR family. The CD40-CD40L complex plays important roles in the functions of CD8^+^ cytotoxic T lymphocytes in innate immune responses and is also necessary for adaptive immune responses [29, 30]. In the present study, TTVAC7 and TTVC5 induced BALB/C mice to generate high levels of VTT-specific CD3^+^CD4^+^/CD8^+^ cells expressing IFN-γ/IL-4 and stimulate the differentiation and maturation of bone marrow dendritic cells, as determined by flow cytometry detection of CD11c^+^/CD80^+^/CD86^+^/CD40^+^/MHC II^+^. Moreover, TTVAC7 and TTVC5 induced rabbits to generate high levels of IFN-γ, IL-6, and TNF-α. These results demonstrate that TTVAC7 and TTVC5 provide excellent immunogenicity.

The complete nucleotide sequences of VTT (GenBank: AF095689.1) and MPXV (GenBank: PP778666.1) share 98% percent identity. VTT has been used to assess the protective effect of vaccines in mice [31, 32]. This mouse model has the following advantages: (1) weight loss of > 25%; (2) pulmonary pathological changes; (3) infectious virus particles detected in lung tissues within 9 days after challenge; and (4) convenient operations and low cost, especially for large-scale experiments. However, this model also has some disadvantages: (1) no skin lesion; (2) 10–30% mortality; (3) disappearance of infectious virus particles at dpi 11; (4) normal body weight restored on dpi 21; and (5) only suitable for preliminary evaluations of vaccines and drugs. In this study, the model group showed remarkable weight loss (up to 32%) from dpi 1 to 14. The body weights of mice immunized with TTVAC7 and TTVC5 were reduced by up to 11.45% and 13.55%, respectively, after VTT challenge. Damage to lung tissues caused by VTT was obviously reduced in mice immunized with TTVAC7 and TTVC5.

The protective effects of TTVAC7 and TTVC5 were also assessed using New Zealand rabbits challenged with MPXV. The benefits of this rabbit model include: (1) skin eruption all over the body; (2) pathological changes to the lungs and testes; (3) infectious virus particles is detectable in skin lesions, lungs, and testes within 6 days after challenge; (4) convenient operations and low cost compared to monkey models, and (5) suitability for evaluation of vaccines and drugs against MPXV. The rabbit model reflects the main symptoms of MPXV infection, such as fever, rash, lesions, pneumonia, and particularly functional damage to the male reproductive system [33–35]. While, the disadvantages of this rabbit model are the disappearance of skin lesions and viral DNA on dpi 10. In this study, the model group developed a high fever of up to 40.3 °C on dpi 5. The body temperatures of rabbits immunized with TTVAC7 and TTVC5 increased to as high as 39.8℃ and 39.6℃, respectively, on dpi 5, although there were fewer pocks, less serious injury to the lungs and testes, and a lower viral titer.

Studies have found that most Chinese populations still maintain VTT-specific IgG antibodies for 42 or more years after smallpox vaccination and could provide some levels of protection against MPXV [36], and some VTT vaccinees showed pre-existing T cell responses to MPXV-derived proteins [37]. In this study, we evaluated the immunogenicity and protective effects of the gene-deleted VTT vaccine. The deleted genes included TC7L-TK2L, TE, TI4L, TJ2R, TA35R, TA66R, and TB13R. The TC7L-TK2L gene fragment is involved in regulating pathogenicity, virulence, and host range of the gene. NYVAC, as a highly attenuated strain of vaccinia virus, was constructed by knockout of this region [38]. TE gene is a host range, virulence and immunomodulatory gene. The protein encoded by TE can suppress the interferon-induced pathway activation [39]. TA35R gene is a virulence and immunomodulatory gene. It can regulate the adaptive immune response. Research indicated deletion of TA35R can reduce viral replication capacity and virulence [40]. TB13R is a host range, virulence and immunomodulatory gene. It has anti-apoptotic and anti-inflammatory effects and has sequence homology with serpins [41]. TB13R encodes non-essential protein which contributes to virus virulence in vivo and affects the host response to infection [42]. TA66R is a virulence gene. It can encode a viral hemagglutinin and inhibits cell fusion [15]. TI4L is a virulence gene which encodes a protein that correlates with neurovirulence [43]. TJ2R encodes a thymidine kinase as an endogenous selection marker of the recombinant vaccinia virus [44]. The deletion of these genes can obviously improve the safety of VTT. While the deletion of these genes also makes host immune system easily identify and clear TTVC5 and TTVAC7. Due to lack of antigen continuous stimulation, immunogenicity and protective efficacy in VTT group were lightly better than those in TTVAC7 and TTVC5 groups.

Nonetheless, the neutralizing antibody titers of the TTVAC7 and TTVC5 groups were markedly increased. The concentration of serum antibodies needed to reduce the number of plaques by 50% [16] against MPXV reached 1:140 and 1:181 in the TTVAC7 and TTVC5 groups, respectively, on dpi 28. A single dose of TTVAC7 and TTVC5 reduced fever, skin rashes, and pathological changes to the lungs and testes of New Zealand rabbits infected with MPXV. Although The deletion of multiple gene may affect the immunogenicity and protective efficacy of VTT. Compared to VTT, TTVAC7 and TTVC5 have shown superior safety and Vaccination with TTVAC7 and TTVC5 protected the New Zealand rabbits against MPXV infection. Moreover, the great advantages of TTVC7 and TTVC5 include: (1) A single dose and (2) simple and easy preparation.

According to the Africa Centers for Disease Control and Prevention, more than 17,000 suspected mpox infections and 517 deaths occurred throughout Africa this year, accounting for an increase of 160% from last year. Mpox remains a public health emergency of international concern. Vaccination is considered the most promising strategy for control of mpox. Hence, the reserve of mpox vaccine should be expanded for clinical application.

## Data Availability

No datasets were generated or analysed during the current study.

## Abbreviations

MPXV: Monkeypox virus
VACV: Vaccinia virus
VTT: VACV strain Tian Tan
IFN-γ: Interferon-γ
IL-6: Interleukin-6
TNF-α: Tumor necrosis factor-α
PE: Phycoerythrin
APC: Allophycocyanin
FITC: Fluorescein isothiocyanate
PerCP: Peridinin chlorophyll protein complex
ELISA: Enzyme-linked immunosorbent assay
Ig: Immunoglobulin
